# BEST4^+^ cells in the intestinal epithelium

**DOI:** 10.1152/ajpcell.00042.2024

**Published:** 2024-04-01

**Authors:** Tania Malonga, Nathalie Vialaneix, Martin Beaumont

**Affiliations:** ^1^GenPhySE, Université de Toulouse, INRAE, ENVT, 31326, Castanet Tolosan, France; ^2^Université de Toulouse, INRAE, UR MIAT, Castanet-Tolosan, France; ^3^Université de Toulouse, INRAE, BioinfOmics, GenoToul Bioinformatics Facility, Castanet-Tolosan, France

**Keywords:** absorptive cells, bestrophin 4, intestinal epithelium, organoids, single-cell transcriptomics

## Abstract

The recent development of single-cell transcriptomics highlighted the existence of a new lineage of mature absorptive cells in the human intestinal epithelium. This subpopulation is characterized by the specific expression of Bestrophin 4 (BEST4) and of other marker genes including *OTOP2, CA7, GUCA2A, GUCA2B*, and *SPIB*. BEST4^+^ cells appear early in development and are present in all regions of the small and large intestine at a low abundance (<5% of all epithelial cells). Location-specific gene expression profiles in BEST4^+^ cells suggest their functional specialization in each gut region, as exemplified by the small intestine-specific expression of the ion channel CFTR. The putative roles of BEST4^+^ cells include sensing and regulation of luminal pH, tuning of guanylyl cyclase-C signaling, transport of electrolytes, hydration of mucus, and secretion of antimicrobial peptides. However, most of these hypotheses lack functional validation, notably because BEST4^+^ cells are absent in mice. The presence of BEST4^+^ cells in human intestinal organoids indicates that this in vitro model should be suitable to study their role. Recent studies showed that BEST4^+^ cells are also present in the intestinal epithelium of macaque, pig, and zebrafish and, here, we report their presence in rabbits, which suggests that these species could be appropriate animal models to study BEST4^+^ cells during the development of diseases and their interactions with environmental factors such as diet or the microbiota. In this review, we summarize the existing literature regarding BEST4^+^ cells and emphasize the description of their predicted roles in the intestinal epithelium in health and disease.

**NEW & NOTEWORTHY** BEST4^+^ cells are a novel subtype of mature absorptive cells in the human intestinal epithelium highlighted by single-cell transcriptomics. The gene expression profile of BEST4^+^ cells suggests their role in pH regulation, electrolyte secretion, mucus hydration, and innate immune defense. The absence of BEST4^+^ cells in mice requires the use of alternative animal models or organoids to decipher the role of this novel type of intestinal epithelial cells.

## Graphical abstract

**Figure:**
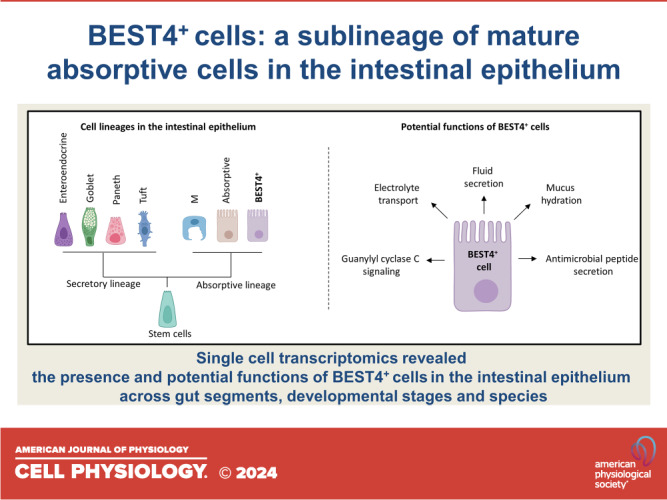

## INTRODUCTION

The intestinal epithelium is a monolayer of cells involved in nutrient uptake while forming a physical and immunological barrier against pathogens and toxins (1). This dual function of the intestinal epithelium relies on diverse types of specialized cells that all derive from stem cells located at the crypt base (2). Progenitors cells differentiate toward the secretory lineage in the absence of Notch signaling. Then, activation of transcription factors further specifies secretory sublineages, including enteroendocrine cells producing hormones, goblet cells secreting mucus, Paneth cells releasing antimicrobial peptides, and Tuft cells playing an important role against parasitic infections (2, 3). Conversely, Notch activation in progenitor cells induces the differentiation of absorptive cells. They represent 80% of epithelial cells in the intestine and are mainly responsible for transport of nutrients, electrolytes, and fluids (2, 3). Absorptive epithelial cells are generally considered a homogeneous population within each gut segment, apart from the rare population of microfold (M) cells localized in the follicle-associated epithelium and that are responsible for antigen sampling (4).

Recent discoveries revealed that the cellular composition of the intestinal epithelium is more complex than previously thought. For instance, a new cell lineage sharing similarities with both Paneth and goblet cells named deep secretory cells was identified in the colon epithelium (5). Spatial transcriptomics also revealed the functional heterogeneity of absorptive cells across the intestinal villus axis (6). The development of single-cell RNA-sequencing (scRNA-Seq) provided an unprecedented resolution to characterize the cellular composition of the intestinal epithelium, as illustrated by the description of goblet cell diversity in the human colon (7). Moreover, scRNA-Seq highlighted a previously overlooked cellular subset of the absorptive lineage in the intestinal epithelium specifically expressing the ion channel Bestrophin 4 (BEST4) (8). In this review, we present the current state of knowledge on BEST4^+^ cells, their localization in the intestinal tract across time and species, the factors potentially involved in their specification in vivo and in organoids, and their potential roles in epithelial physiology in health and disease.

## BEST4^+^ INTESTINAL EPITHELIAL CELLS ACROSS GUT SEGMENTS, DEVELOPMENT AND SPECIES

### Identification of BEST4^+^ Cells in the Intestinal Epithelium

In 2013, the seminal study by Ito and colleagues used immunohistochemistry to describe a subpopulation of human absorptive epithelial cells that specifically express BEST4, a calcium-sensitive ion channel transporting bicarbonate (HCO_3_^−^) and chloride (Cl^−^) ions (8). The existence of BEST4^+^ cells was then confirmed a few years later with the advent of scRNA-Seq, which allowed the creation of single-cell transcriptomic atlases of the human intestinal epithelium (7, 9). Epithelial cell clustering based on transcriptomic profiles indicated that human BEST4^+^ cells express a unique set of genes including *BEST4*, Otopetrin 2 (*OTOP2*), Guanylate cyclase activator 2A (*GUCA2A*), Guanylate cyclase activator 2B (*GUCA2B*), Carbonic anhydrase 7 (*CA7*), and Spi-B transcription factor (*SPIB*) (7, 9–19). The presumed role of these genes in BEST4^+^ cells will be presented in the following sections.

Due to their recent discovery, diverse names were used in scRNA-Seq studies to label this cell population, such as “Paneth-like” cells (20) or SPIB^+^ cells (14) or CA7^+^ cells (21) or BEST4/OTOP2 cells (B/OC) (22). The expression of *SPIB* by BEST4^+^ cells led some authors to consider them as microfold (M) cells (23) since the Spi-B transcription factor has mainly been described for its role in the maturation of M cells (4). A subpopulation of small intestinal epithelial cells expressing high levels of the cystic fibrosis transmembrane conductance regulator (*CFTR*) previously known as CFTR High Expresser (CHE) cells was found later in humans to also express the typical markers of BEST4^+^ cells and was therefore renamed BCHE cells (24, 25). In this review, for clarity, we will only use the name BEST4^+^ cells to refer to the subpopulation of epithelial cells expressing the canonical markers described earlier.

### BEST4^+^ Cells across Species

**Figure 1.**
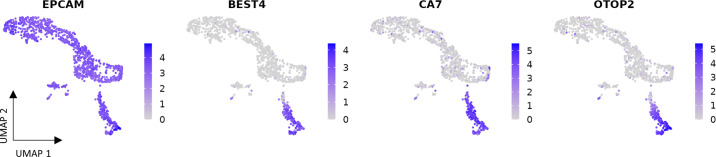
Single-cell RNA-sequencing reveals the presence of BEST4^+^ cells in the rabbit caecum epithelium. Epithelial cells from the cecum of a 27-day-old male rabbit were isolated and processed for single-cell RNA-sequencing using Chromium Next GEM Single Cell 3′ Reagent Kits v3.1 (10xGenomics). Data processed with the Cell Ranger software (10x Genomics) are available at https://doi.org/10.57745/0NUYZR. Seurat pipeline analysis scripts and results are also available in the same repository. Single-cell transcriptome profiles are shown by uniform manifold approximation and projection (UMAP) visualizations colored according to the gene expression level of the epithelial marker *EPCAM* (Epithelial Cell Adhesion Molecule) and of BEST4^+^ cells canonical makers: *BEST4* (Bestrophin 4), *CA7* (Carbonic Anhydrase 7), and *OTOP2* (Otopetrin 2).

**Figure 2.**
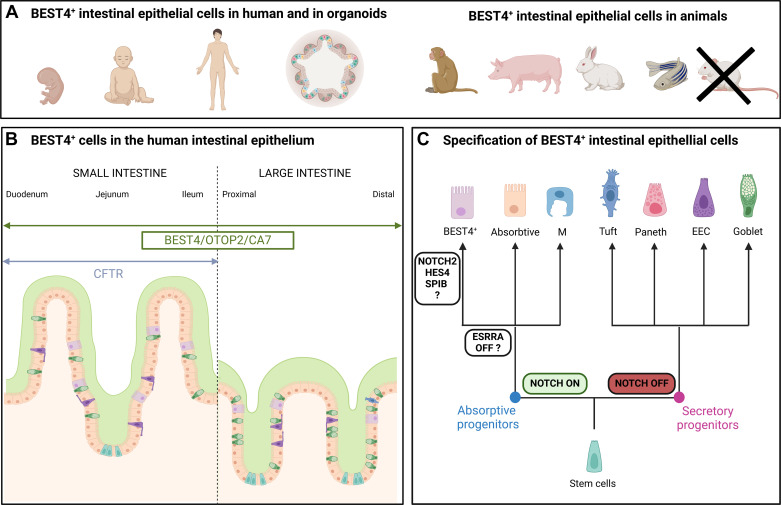
BEST4^+^ cells in the intestinal epithelium. *A*: BEST4^+^ cells are present in the human intestinal epithelium, appear early in development and can be found in organoids. BEST4^+^ cells were described in the intestinal epithelium of monkey, pig, rabbit, zebrafish, but not in mice. *B*: BEST4^+^ cells express bestrophin 4 (*BEST4*), otopetrin 2 (*OTOP2*), and carbonic anhydrase 7 (*CA7*) in all segments of the small and large intestine while cystic fibrosis transmembrane conductance regulator (*CFTR*) is expressed specifically in the small intestine. BEST4^+^ cells are mostly found in villi in the small intestine and at the upper part of crypts in the large intestine. BEST4^+^ cells are often located near goblet cells, which is consistent with the potential role of BEST4^+^ cells in mucus hydration. *C*: BEST4^+^ cells are a sublineage of mature absorptive cells. Factors potentially involved in the specification of BEST4^+^ cells from absorptive progenitors include estrogen-related receptor α (ESRRA), Spi-B Transcription Factor (SPIB), Hes Family BHLH Transcription Factor 4 (HES4), and Notch Receptor 2 (NOTCH2). EEC, enteroendocrine cell. Figure created with Biorender.com.

Several scRNA-Seq studies showed that BEST4^+^ cells are not present in the intestine of mice (21, 26–28). Indeed, *BEST4* was identified as a pseudogene in the mouse genome, suggesting that the functional version of this bestrophin paralog was lost in this species (29). Interestingly, other genes known to be specifically expressed by intestinal BEST4^+^ cells in the intestines of other species (e.g., *OTOP2*, *CA7*, and *GUCA2A*) are present in the mouse genome. This suggests that the *BEST4* gene may be crucial for the establishment and functions of this subtype of mature absorptive cells in the intestinal epithelium. In contrast with mice, a functional *BEST4* gene is present in the rat genome (29). However, a scRNA-Seq study did not find a BEST4^+^ population in the rat ileum epithelium (21). BEST4^+^ cells may be present in other digestive segments since the presence of CHE cells was demonstrated in the rat proximal small intestine (30). Yet, the equivalence between CHE cells and BEST4^+^ cells was only demonstrated in the human small intestine (24) and should be confirmed in rats. However, BEST4^+^ cells are not specific to the human intestinal epithelium since cell populations expressing the same makers (*BEST4, GUCA2A, GUCA2B, OTOP2,* and *CA7*) were identified in the intestine of pigs (21, 22, 31, 32) and macaques (21). Yet, the transcriptomic profile of BEST4^+^ cells seemed less conserved between species, when compared with other types of epithelial cells (21). Our scRNA-Seq analysis of rabbit cecum epithelial cells also indicated the presence of a population of BEST4^+^ cells characterized by the expression of *BEST4, CA7,* and *OTOP2* (Fig. 1). Interestingly, a population of enterocytes expressing *BEST4* and *OTOP2* were also identified in the zebrafish intestine (33). Overall, the presence of BEST4^+^ cells in the intestine of vertebrates seems evolutionarily conserved and their absence in mice might be an exception (Fig. 2*A*).

### BEST4^+^ Cells in the Developing Intestinal Epithelium

BEST4^+^ cells are found in the human intestinal epithelium at embryonic, fetal, pediatric, and adult stages (7, 10–12, 19, 23, 34) (Fig. 2*A*). BEST4^+^ cells are already present in the developing human intestine at 11 postconceptional weeks, before the formation of crypts, which is in contrast to enteroendocrine cells, goblet cells, and mature enterocytes (12). BEST4^+^ cells represent less than 5% of epithelial cells in the fetal intestine and their abundance remains the same during in utero development (11, 12), whereas the proportions of goblet and enteroendocrine cells tend to rise from 12 to 19 postconceptual weeks in humans (12). A study in piglets showed that the abundance of BEST4^+^ cells (5%–10%) remained stable in the ileum across the suckling-to-weaning transition (22), which is a major step for the postnatal maturation of the intestinal epithelium. Altogether, these data suggest that BEST4^+^ cells are established early in utero and their abundance seems stable during intestinal development in human and pigs, although this developmental pattern should be confirmed by additional studies and validated in other species.

### Localization of BEST4^+^ Cells in the Crypt-Villus Axis

Immunohistochemistry labeling and RNA in situ hybridization showed that human BEST4^+^ cells are mostly located in villi (small intestine), on the surface epithelium and on the crypt top (small and large intestine) (8, 9, 15) (Fig. 2*B*). In pigs, BEST4^+^ cells are present both in the upper part of crypts and in villi throughout the small intestine (31). The BEST4^+^ cells marker CA7 was also distributed in villi of pigs and macaque ileum (21). Interestingly, BEST4^+^ cells tend to neighbor goblet cells in human and pigs (8, 10, 31), which is consistent with a possible contribution of BEST4^+^ cells to the hydration of mucus, as presented in *Electrolyte Transport and Mucus Hydration*. Expression of marker genes of epithelial cell position in the crypt-axis predicted BEST4^+^ cells to be located between the middle and the top of epithelial crypts (7, 10, 14, 17, 35). Spatial transcriptomics performed on sections of human colon also localized BEST4^+^ cells toward the top of epithelial crypts and highlighted their colocalization with mature colonocytes (12). All studies agree that BEST4^+^ cells are never seen in the bottom of the crypts where proliferative and epithelial stem cells are located. Accordingly, BEST4 protein does not colocalize with the proliferation marker *K*_i_-67 in the human intestinal epithelium (8), which indicates that BEST4^+^ cells are postmitotic.

### Regional Differences in BEST4^+^ Cells

BEST4^+^ cells are found in each segment of the small and large intestine (8, 15, 18, 19). In human adults, the abundance of BEST4^+^ cells represents 5%–15% of the total epithelial population in the jejunum, ileum, and colon while they represent less than 2% in the duodenum, appendix, and rectum (11, 24). However, there are some variations between studies since others found that BEST4^+^ cells represent only 1% to 3% of cells in human ileum (16, 21) and testing for differential abundance of BEST4^+^ cells according to gut location revealed no significant changes despite the highest proportions observed in the colon (18). Other studies found an abundance lower than 5% for BEST4^+^ cells in the human colon (9, 14). BEST4^+^ cells are also rare (<4%) in pig and macaque ileum (21, 31). Overall, BEST4^+^ cells are low-abundance cells of the intestinal epithelium but additional studies are still required to define more precisely their frequency and distribution across gut segments and species.

Location-specific gene expression profiles were observed in BEST4^+^ cells (small vs. large intestine) in human adults but not in fetal tissues (11) suggesting postnatal acquisition of regionalized functions. The most striking difference is the expression of *CFTR* by BEST4^+^ cells in the small intestine but not in the colon (11, 15, 24, 25) (Fig. 2*B*). Other regional differences include a higher expression of ATP-Binding Cassette Subfamily G Member 5 (*ABCG5*), Neuropeptide Y (*NPY*), lysozyme (*LYZ*), Bone Morphogenetic Protein 3 (*BMP3*), and metallothioneins (*MTs*) in small intestine BEST4^+^ cells while Otopetrin 3 (*OTOP3*) and Secretory Leukocyte Peptidase Inhibitor (*SLPI*) are examples of genes specifically expressed by colon BEST4^+^ cells (11, 15, 24). These observations suggest functional specialization of BEST4^+^ cells in each gut region.

### Specification of BEST4^+^ Cells

The coexpression of BEST4 and villin in human epithelial cells was the first evidence indicating that BEST4^+^ cells belong to the absorptive lineage (8). Moreover, confocal microscopy imaging suggested that the morphology of BEST4^+^ cells is not distinctive from other absorptive cells, with an elongated and columnar shape (8). Yet, electron microscopy studies indicated that the morphology of CHE cells in the rat intestine was slightly distinctive from other mature enterocytes, with less densely packed microvilli and characterized by the accumulation of apical vesicles (25, 36). Further studies would be required to determine if these ultrastructural features of CHE cells are also observed in the analogous BEST4^+^ cells in the intestinal epithelium. Trajectory inference analysis based on scRNA-Seq data from the human small and large intestine epithelium also suggests that BEST4^+^ cells constitute a terminal state that branches from absorptive progenitors and that is distinct from mature enterocytes or colonocytes (9, 14, 17, 35). The branching of BEST4^+^ cells from absorptive progenitors is marked by a reduced gene expression of the transcription factor estrogen-related receptor α (*ESRRA*) when compared with other mature absorptive cells (35) (Fig. 2*C*). In contrast, one study suggested that small intestine BEST4^+^ cells may arise from secretory progenitors but this conclusion may be qualified by the relatively small number of cells analyzed to infer temporal lineage trajectories (15).

BEST4*^+^* cells specifically express the Notch 2 receptor (*NOTCH2*), suggesting that Notch signaling could be involved in their specification to the absorptive lineage (7, 9, 14, 15, 17). Accordingly, the upregulation of BEST4 expression associated with in vitro differentiation of Caco-2 cells requires Notch signaling (8). The transcription factors *HES4* and *SPIB* expressed by BEST4^+^ cells could also play an important role in their differentiation (7, 9, 14, 15, 17). Thus, available data indicate that BEST4^+^ cells constitute a mature subpopulation within the absorptive lineage in the intestinal epithelium but the molecular signals and transcription factors driving their specification remain to be validated experimentally.

### BEST4^+^ Cells in Intestinal Organoids

In vitro models are required to study the specification and the still largely unknown functions of BEST4^+^ cells in the intestinal epithelium. BEST4^+^ cells can be found in human organoids derived from fetal and adult epithelial crypts (10, 34, 37). The proportion of BEST4^+^ cells in organoids derived from the fetal intestine was higher when WNT3A was included in the culture medium in comparison with a growth medium lacking this Wnt ligand (10). Another study showed that replacing epidermal growth factor (EGF) in the culture medium with epiregulin (EREG), another EGF family member, was necessary to observe BEST4^+^ cells in human intestinal organoids derived from fetal epithelial crypts (34). A rare population of cells expressing *BEST4, CA7, SPIB, GUCA2A*, and *GUCA2B* (labeled as M cells) was also present in human intestinal organoids derived from embryonic stem cells and transplanted into the kidney capsule of mice for 8 wk (23). Utilization of available intestinal organoids from other species such as pigs, rabbits, and rats (38–40) could also be useful to study BEST4^+^ epithelial cells but their capacity to retain this rare population remains to be validated. The possibility to study BEST4^+^ cells in vitro by using intestinal organoids that are amenable to genetic modifications opens numerous perspectives for functional studies and to decipher the molecular events driving their specification. However, a limitation of commonly used tissue-derived organoids is the absence of nonepithelial cells, such as mesenchymal cells, immune cells, or neurons, which prevents the use of this model to assess the interactions between BEST4^+^ cells and these specific cell types.

## POTENTIAL FUNCTIONS OF BEST4^+^ CELLS IN THE INTESTINAL EPITHELIUM

Due to their recent discovery, the functions of BEST4^+^ cells remain largely unknown. Current hypotheses are mainly based on ontology analyses of genes expressed specifically by BEST4^+^ cells and on potential cell-cell interactions inferred from ligand-receptor expression in scRNA-Seq data. It is important to note that functional validations are currently lacking for most of the potential functions of BEST4^+^ cells.

### Electrolyte Transport and Mucus Hydration

**Figure 3.**
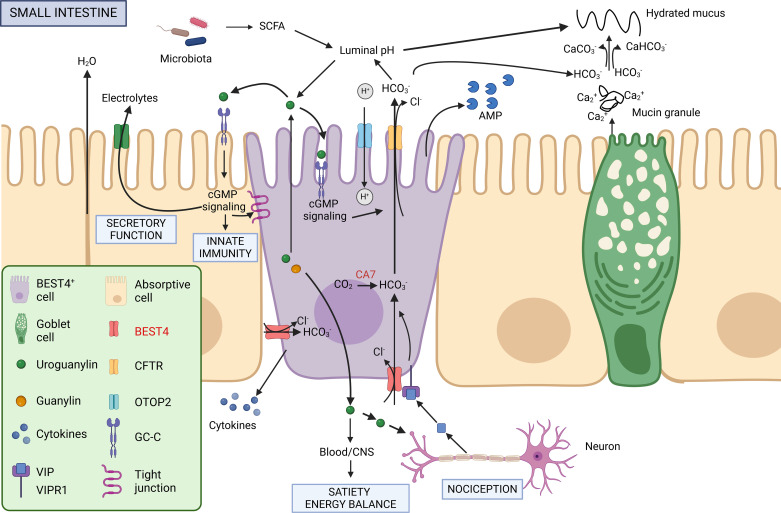
Potential functions of BEST4^+^ cells in the small intestine. The predicted function of BEST4^+^ cells presented in this figure were inferred from their gene expression profile and remain to be validated experimentally. Bicarbonate (HCO_3_^−^) and chloride (Cl^−^) can be transported by the Bestrophin 4 (BEST4) ion channel expressed at the basolateral side of BEST4^+^ cells. Carbonic anhydrase 7 (CA7) can also contribute to produce HCO_3_^−^ in BEST4^+^ cells by hydration of CO_2_. HCO_3_^−^ and Cl^−^ can be secreted in the lumen by the ion channel cystic fibrosis transmembrane conductance regulator (CFTR), which is highly expressed by BEST4^+^ cells in the small intestine. Ion secretion by BEST4^+^ cells could lead to water secretion, hydration of mucus, and regulation of luminal pH. In turn, BEST4^+^ cells can respond to changes in luminal pH by importing protons though Otopetrin 2 (OTOP2), which is able to regulate intracellular pH in BEST4^+^ cells. Ion transport by BEST4^+^ cells could also be tuned by activation of the vasoactive intestinal peptide receptor (VIPR1) or by Guanylate cyclase-C receptor (GC-C receptor) signaling. Indeed, BEST4^+^ cells can release guanylin (GUCA2A) and uroguanylin (GUCA2B) into the lumen and activate the GC-C receptor, which is expressed by most epithelial cell types. Subsequently, activation of cGMP signaling regulates electrolyte secretion, cellular junctions, and innate immunity. In BEST4^+^ cells, an autocrine-loop involving pH sensing and GC-C/c-GMP signaling could be involved in the regulation of ion secretion and production of antimicrobial peptides (AMP) targeting the gut microbiota. Finally, BEST4^+^ cells could regulate nociception, satiety, and energy balance through the secretion of guanylin and uroguanylin at the basolateral side. CNS, central nervous system; SCFA, short-chain fatty acids. Figure created with Biorender.com.

Genes expressed by BEST4^+^ cells suggest their important role in transepithelial transport of electrolytes and fluid secretion (7, 9, 21) (Fig. 3). Bestrophins constitute a family of four paralogs (*BEST1, BEST2, BEST3*, and *BEST4*) coding calcium-activated chloride channels expressed at the basolateral membrane of epithelial cells (41). Bestrophins have a broad tissue distribution and are expressed in the epithelium of multiple organs including the retina, lung, colon, pancreas, kidney, and testis (42). Bestrophins mediate the flow of chloride and other monovalent anions (e.g., HCO_3_^−^) across cell membranes, thereby influencing fluid secretion and cell volume (41–43). *BEST1* is the most studied paralog since numerous mutations in this gene are involved in human retinal degenerative disorders linked to abnormal fluid and electrolyte transport (41). Mouse BEST2 is involved in bicarbonate transport by colon goblet cells (44). Although there has been limited attention given to the specific functions of the *BEST4* paralog, it can be presumed that BEST4 expressed at the basolateral membrane of BEST4^+^ cells may import bicarbonate and chloride into the cytosol (8, 45). CA7 could also contribute to the production of bicarbonate in BEST4^+^ cells by catalyzing hydration of CO_2_ (46). At the apical membrane of small intestine BEST4^+^ cells, CFTR can secrete bicarbonate and chloride into the intestinal lumen, thereby creating an osmotic gradient driving fluid secretion (25). Other ion channels might play this role in large intestine BEST4^+^ cells that do not express CFTR (15). Secretion of bicarbonate by BEST4^+^ cells could then contribute to the formation of the mucus layer by binding calcium in mucin granules and by hydrating mucus (47). This potential role of BEST4^+^ cells in the formation of mucus is consistent with their frequent proximity with goblet cells in the intestinal epithelium (8, 10, 31).

### Response to Luminal pH and Regulation of cGMP Tone

Secretion of bicarbonate mediated by BEST4^+^ cells could contribute to regulating the luminal pH, which has a key role in digestive processes and prevention of pathogen growth while providing an appropriate environment for the commensal microbiota (25). Interestingly, in vitro experiments showed that sorted BEST4^+^ epithelial cells are able to conduct protons into their cytosol when extracellular pH decreases (7). The ability of BEST4^+^ cells to sense and respond to pH changes could be mediated by the proton-conducting pH-sensitive ion channel OTOP2 (48). This potential regulation of pH by BEST4^+^ cells could modulate the guanylate cyclase-C receptor (GC-C)/cyclic guanosine monophosphate (cGMP) signaling pathway, which is pH-sensitive (49), and which ligands (uroguanylin and guanylin, coded by *GUCA2A* and *GUCA2B*) are highly expressed by BEST4^+^ cells. Binding of these paracrine/autocrine hormones to the apical GC-C receptor (GUCY2C), which is expressed by most intestinal epithelial cell types including BEST4^+^ cells (15), increases the production of cGMP (49). cGMP is a secondary messenger that regulates transepithelial fluid movements through the activation of ion channels such as CFTR. This could trigger an autocrine loop in BEST4^+^ cells leading to electrolytes and fluid secretion (24). The potential modulation of intestinal cGMP tone by BEST4^+^ cells may be key for intestinal homeostasis since the cGMP signaling pathway regulates cellular proliferation, epithelial barrier function, inflammation, and visceral nociception (49).

### Neuroendocrine Regulations

The neuropeptides Vasoactive Intestinal Peptide (VIP) and NPY could regulate the secretory functions of BEST4^+^ cells (50). Indeed, receptor-ligand analysis based on scRNA-Seq data predicted that VIP secreted by inhibitory motor neurons could modulate secretory functions of BEST4^+^ cells that express the VIP receptor *VIPR1* (9, 12). The expression of *NPY* by small intestine BEST4^+^ cells might also regulate their secretory function (50) and mediate a crosstalk with enteroendocrine cells that express the NPY receptor (*NPY1R*) (15). BEST4^+^ cells could also modulate satiety and energy balance through the secretion of uroguanylin, which can be transported into the central nervous system via the bloodstream where it binds to the GC-C receptor in the hypothalamus (51). Moreover, regulation of intestinal motility by BEST4^+^ cells was also predicted based on their expression of *NPY*, Adrenoceptor α 2A (*ADRA2A*), and Cholinergic Receptor Muscarinic 3 (*CHRM3*) (9, 15).

### Epithelial Defenses

BEST4^+^ cells may play an important role in the antimicrobial defenses of the intestinal epithelium. Indeed, BEST4^+^ cells express antimicrobial peptides including lysozyme (*LYZ*), serine protease 3 (*PRSS3*), defensin α 5 (*DEFA5*), and phospholipase A2 group IIA (*PLA2G2A*), LY6/PLAUR Domain Containing 8 (*LYPD8*), Deleted In Malignant Brain Tumors 1 (*DMBT1*), WAP Four-Disulfide Core Domain 2 (*WFDC2*), and Secretory Leukocyte Peptidase Inhibitor (*SLPI*) (7, 9, 15). The expression of antimicrobial peptides by human BEST4^+^ cells is regionalized since *LYZ* and *DMBT1* are mainly expressed in the small intestine while *LYPD8*, *WFDC2*, and *SLPI* are mainly expressed in the large intestine (15, 19). The antimicrobial peptides expressed by BEST4^+^ cells are also produced by other epithelial cell types (notably Paneth and Tuft cells), suggesting overlaps in their antimicrobial activities (15, 19).

In addition, BEST4^+^ cells highly express genes from the metallothionein (MT) family including *MT1E, MT1G, MT1H, MT1M, MT1X*, and *MT2A* (7, 9, 15, 23, 35, 52, 53). The key role of MT in metal-ion homeostasis and in the regulation of immune and oxidative responses in the intestine (54) could provide BEST4^+^ cells an essential role in the epithelial barrier function. BEST4^+^ cells may also interact with immune cells through the expression of genes coding for cytokines (e.g., *CCL23, CCL15*, and *IL18*) (9). Similarly to other epithelial cells, BEST4^+^ cells are able to mount a type I interferon response upon viral infection, as demonstrated in human intestinal organoids infected by Human astrovirus 1 (37). Therefore, BEST4^+^ cells could play an essential role in the preservation of epithelial integrity.

### BEST4^+^ Intestinal Epithelial Cells in Inflammatory Bowel Diseases

A few studies suggested that BEST4^+^ cell homeostasis could be disrupted in inflammatory bowel diseases (IBD), including ulcerative colitis (UC) and Crohn’s disease (CD). A scRNA-Seq study reported a slightly reduced abundance of BEST4^+^ cells in inflamed colon biopsies from patients with UC when compared with healthy controls (9). However, this depletion of BEST4^+^ cells in UC was not seen in other studies using scRNA-Seq (7) or immunochemistry (8). The two scRNA-Seq studies similarly found that the expression level of MTs was reduced in colon BEST4^+^ cells from patients with UC (7, 9). Other genes differentially expressed in colon BEST4^+^ cells between healthy patients and patients with UC coded for antimicrobial peptides and cytokines but were not shared between the two studies (7, 9). In CD, a study found that the proportion of BEST4^+^ cells was reduced in the ileum of treatment naïve or established patients (16). In contrast, another study found a higher proportion of mature BEST4^+^ cells (i.e., expressing *OTOP2*) in the colon epithelium of patients with CD (14). In pediatric CD, the abundance of BEST4^+^ cells in the ileum was similar to healthy controls (10). Overall, some evidence suggest that homeostasis of BEST4^+^ cells might be altered in IBD but additional studies are clearly required to confirm these findings since contradictory results were obtained, as illustrated by a comparative analysis (53).

## PERSPECTIVES TO UNRAVEL THE PHYSIOLOGY OF BEST4^+^ CELLS IN THE INTESTINAL EPITHELIUM

The recent discovery of BEST4^+^ cells in the intestinal epithelium highlighted a previously neglected heterogeneity in the absorptive lineage. The potential functions of BEST4^+^ cells (cGMP signaling, pH regulation, electrolyte and fluid transport, and antimicrobial defenses) suggest that they may be central in epithelial homeostasis and that they could be potentially involved in multiple diseases including IBD, visceral pain, diarrheal diseases, cystic fibrosis, and obesity (7, 25, 49). However, the absence of BEST4^+^ cells in the mouse intestine has hampered the functional studies required to explore the pathways driving their differentiation and their role in health and disease. The presence of BEST4^+^ cells in human intestinal organoids represents a major opportunity to study the specification of this novel sublineage of absorptive epithelial cells by modulating key signaling pathways (e.g., Notch, Wnt) or by invalidating transcription factors potentially involved in the differentiation of BEST4^+^ cells (e.g., *SPIB*, *HES4*, and *ESRRA*). Invalidation of genes specifically expressed by BEST4^+^ cells (e.g., *BEST4*, *OTOP2*) in human intestinal organoids would also be instrumental in understanding their role in secretory, absorptive, and barrier function of the intestinal epithelium. Studies in animal models (e.g., rabbit, pig, zebrafish) are also required to decipher the interaction of BEST4^+^ cells with nonepithelial cell types (e.g., immune cells, neurons), their role in diseases, and the mechanisms involved in their pre- and postnatal development in interaction with environmental factors such as nutrition or the gut microbiota (e.g., by using germ-free animals). For instance, it can be hypothesized that BEST4^+^ cells could sense and respond to modulations of luminal pH associated with the bacterial production of short-chain fatty acids. Overall, a greater understanding of the role of BEST4^+^ intestinal epithelial cells in health and diseases is expected in the coming years thanks to additional single-cell examination of the intestinal epithelium in humans and in animal models combined with in vitro reductionist experiments in organoids.

## GRANTS

This work was supported by a grant from the French National Research Agency: ANR-JCJC MetaboWean (ANR-21-CE20-0048).

## DISCLOSURES

No conflicts of interest, financial or otherwise, are declared by the authors.

## AUTHOR CONTRIBUTIONS

T.M. and N.V. analyzed data; T.M. prepared figures; T.M. drafted manuscript; T.M., N.V., and M.B. edited and revised manuscript; T.M., N.V., and M.B. approved final version of manuscript.
